# Health-related quality of life in esophageal cancer: a state-of-the-art review of patient-reported outcomes and an evidence and gap map

**DOI:** 10.1093/dote/doaf086

**Published:** 2025-10-12

**Authors:** Kenneth Färnqvist, Kalle Mälberg, Sophie I Johnsson, Asif Johar, Anna Schandl, Cecilia Ringborg, Pernilla Lagergren

**Affiliations:** Department of Molecular Medicine and Surgery, Karolinska Institute, Stockholm, Sweden; Department of Molecular Medicine and Surgery, Karolinska Institute, Stockholm, Sweden; Department of Molecular Medicine and Surgery, Karolinska Institute, Stockholm, Sweden; Department of Molecular Medicine and Surgery, Karolinska Institute, Stockholm, Sweden; Department of Molecular Medicine and Surgery, Karolinska Institute, Stockholm, Sweden; Department of Perioperative and Intensive Care, Södersjukhuset, Stockholm, Sweden; Department of Clinical Science and Education, Södersjukhuset, Karolinska Institute, Stockholm, Sweden; Department of Molecular Medicine and Surgery, Karolinska Institute, Stockholm, Sweden; Department of Molecular Medicine and Surgery, Karolinska Institute, Stockholm, Sweden; Department of Surgery & Cancer, Imperial College London, London, UK

**Keywords:** cancer, esophageal, interventions, patient-reported outcomes, stomach neoplasm, symptoms

## Abstract

Esophageal cancer represents a substantial global health challenge, marked by poor prognosis, even after curative treatment. Health-related quality of life is crucial for evaluating the treatment efficacy and long-term outcomes in patients with esophageal cancer. This state-of-the-art review and evidence gap map sought to identify existing research on the impact of interventions on health-related quality of life in adults with esophageal cancer, providing a comprehensive overview of various health-related quality of life aspects following diagnosis and treatment. This review entailed a systematic literature search, data extraction, and analysis, with the findings visualized in an evidence and gap map. The review synthesized key insights from the literature, focusing on clinical context, treatment, health-related quality of life outcomes, and interventions to enhance health-related quality of life. The evidence and gap map revealed that most studies concentrated on surgical interventions, chemotherapy/chemoradiotherapy, supportive care, and lifestyle interventions, primarily evaluating the overall quality of life, symptom burden, and emotional and psychological health. Several areas remain unexplored, including cognitive and existential well-being, social functioning, and the impact of specific interventions such as immunotherapy. This review underscores the need for high-quality longitudinal studies assessing long-term health-related quality of life, the inclusion of health-related quality of life as a primary or key secondary endpoint in future trials, and improved methodological quality of systematic reviews. Addressing these gaps will contribute to a more patient-centered, evidence-based approach to esophageal cancer care.

## INTRODUCTION

In Franz Kafka’s *A Hunger Artist* (1922), the protagonist undergoes a prolonged fast, serving as both a spectacle and a form of self-imposed penance. Ultimately, he dies in obscurity, unrecognized and unfulfilled. His voluntary abstinence from food, once celebrated and later forgotten, symbolizes existential suffering, physical decline, and profound alienation. Patients with esophageal cancer do not choose to abstain from food; their profoundly impaired ability to eat is imposed by a devastating disease and requires surgical treatment, resulting in a physiological obstruction that disrupts one of the most fundamental human rituals. For patients with esophageal cancer, the impaired eating ability is not a choice but a consequence of the disease itself, a physiological obstruction that disrupts one of the most fundamental human rituals. As advancements in treatment have extended survival rates, the focus has increasingly shifted toward the human cost of survival, particularly the ongoing challenges related to swallowing, speech, social engagement, and identity. In this context, health-related quality of life (HRQL) is not merely a secondary outcome but a central concern, encompassing the physical, emotional, and existential dimensions of living with and beyond esophageal cancer.

### Rationale for the evidence and gap map

Historically, esophageal cancer treatment outcomes have primarily been assessed based on survival rates and oncological effectiveness. In recent years, there has been growing recognition of the importance of incorporating HRQL in the assessment of treatment outcomes.^1^^,^^2^ This review of evidence and gaps aimed to identify available research related to the effects of interventions on HRQL in adults with esophageal cancer. The interventions could have been applied anywhere during the cancer journey, including survivors undergoing an intervention. Furthermore, it will provide a state-of-the-art resume regarding various HRQL aspects following esophageal cancer diagnosis and treatment, including recovery time, factors influencing HRQL, and long-term HRQL outcomes. This review will help pinpoint the available evidence, including the quality of evidence synthesis, and identify gaps, thereby informing future research in this field.

## METHODS

Given the substantial annual increase in published articles,^3^ maintaining current knowledge and determining research priorities have become increasingly challenging. One approach to address this issue is to create evidence and gap maps (EGMs). Systematic mapping of existing evidence in a particular domain enables the identification of knowledge gaps and highlights areas where future research efforts should be concentrated.^4^^,^^5^ This work is reported as an interpretive synthesis based on the results of a systematic search, with an embedded evidence and gap map to visualize the distribution of available evidence and identify research gaps.^6^ The process included formulating a research question, establishing inclusion and exclusion criteria, conducting a systematic literature search, extracting and analyzing the data, and visualizing the results. Furthermore, the methodology employs rapid approaches at specific stages according to the recommendations provided by the Cochrane Rapid Reviews Methods group.^7^ Detailed information regarding the search strategy, screening, coding, and synthesis is provided in Supplementary material 1.

In addition to systematic mapping, an interpretive literature review, informed by expert opinions, was conducted to contextualize the evidence base and synthesize key insights pertinent to clinical practice and future research. While the majority of references cited in the narrative section were identified through a systematic search (the same search as for the EGM), selected practice-changing studies were included irrespective of their publication date. Although the review aimed to prioritize the literature published in recent years, the inclusion of landmark trials and high-impact studies was deemed essential for providing a comprehensive and current overview of HRQL outcomes following the diagnosis and treatment of esophageal cancer.

## THE EXPERT OPINION REVIEW

### Clinical context

Esophageal cancer is a significant global health issue, with approximately 600,000 cases reported in 2020.^8^ The incidence is particularly elevated among men, with more than one in seven cases occurring in the male population.

Accurate diagnosis and staging are essential to determine appropriate therapeutic interventions.^9^^,^^10^ Critical factors influencing treatment decisions include the tumor’s anatomical location, histopathological subtype, clinical and pathological stage, and patient-specific considerations, such as overall health status and comorbidities. Biomarker testing, including HER2, MSI/MMR, PD-L1, and TMB, is increasingly employed to stratify patients and tailor treatment approaches.^11^ Incorporating patient-reported outcome measures (PROMs) into clinical settings yields valuable insights into symptoms and quality of life (QoL), offering supplementary data along with clinical indicators. PROMs can enhance predictive models, facilitate longitudinal monitoring through digital tools, and support treatment strategies that align with patient priorities.^12^

Staging systems and therapeutic strategies for esophageal cancer have evolved to reflect an enhanced understanding of disease progression and treatment response. The eighth edition of the American Joint Committee on Cancer staging manual^10^ incorporates multiple staging time points and emphasizes the role of neoadjuvant therapy. Current clinical guidelines advocate a multidisciplinary approach and support a range of interventions including endoscopic procedures, surgery, and systemic therapy tailored to tumor characteristics and individual risk profiles.^11^^,^^13^ These developments underscore the importance of aligning clinical decisions with both oncological criteria and the values and long-term well-being of patients.^11^

### Treatment

Surgical removal continues to be the primary treatment approach for both localized and locally advanced esophageal cancers,^14^ frequently in conjunction with neoadjuvant and/or adjuvant chemotherapy and/or radiotherapy.^15^ Consequently, there is a considerable risk of postoperative complications.^16^ Curative-intent treatment often combines surgery with neoadjuvant therapy with decisions guided by a multidisciplinary team. There is no universal strategy; the optimal approach depends on tumour location, stage, and histological subtype.^17^ Minimally invasive and robot-assisted techniques are increasingly preferred because of their reduced perioperative morbidity and improved short-term HRQL outcomes without compromising survival.^18–23^ The choice of surgical approach, the transthoracic approach (for example, Ivor Lewis and McKeown) versus the transhiatal approach, remains controversial and is frequently determined by surgeon expertise and patient-specific factors.^24^

Neoadjuvant chemoradiotherapy (nCRT) is the standard treatment for locally advanced disease. In the case of squamous cell carcinoma, nCRT is generally preferred,^25^^,^^26^ whereas both nCRT and perioperative chemotherapy are used for adenocarcinoma.^27–29^ Treatment decisions should be individualized based on the tumor biology and patient conditions. Multiple studies have shown that combining nCRT with surgery improves overall survival rates compared to surgery alone.^30^ Nonetheless, the optimal neoadjuvant regimen remains a topic of discussion, particularly concerning the balance between oncologic efficacy and treatment-related morbidity, which may affect long-term HRQL.^31^ Some evidence indicates that long-term HRQL does not significantly differ between patients receiving nCRT and those undergoing surgery alone.^32^

Approximately 20%–40% of patients diagnosed with esophageal cancer qualify for curative treatment.^33^^,^^34^ Despite surgery with curative intent, long-term survival rates range from 30% to 50% and are often accompanied by significant postoperative complications.^16^^,^^35^ The overall complication rate following esophagectomy is approximately 60%, with pneumonia being the most prevalent adverse event.^36^ In the absence of surgical intervention, the 5-year overall survival rate remains low, typically below 10%.^37^ Recurrence is observed in up to 50% of the patients undergoing curative treatment.^38^ Although the risk diminishes after 5 years,^17^ the 10-year survival rate remains low at approximately 12%.^39^ While the pathological complete response (pCR) is correlated with improved outcomes, one study indicated that even patients achieving pCR following nCRT experienced a 21% recurrence rate within 24 months post-treatment.^40^ A recent randomized controlled trial (RCT) showed that 41% of patients who underwent esophagectomy survived beyond 5 years. Among these survivors, 19% experienced disease recurrence and several developed secondary primary cancers, most frequently in the head, neck, or lungs. These findings challenge the conventional assumption that 5-year survival equates to a cure.^41^

Given the significant morbidity and mortality associated with esophageal cancer, HRQL is crucial for evaluating the treatment efficacy and long-term outcomes. A recent systematic review with meta-analyses identified a statistically significant correlation between global QoL and mortality (hazard ratio [HR] 1.02; 95% CI: 1.01–1.04; *P* < 0.001), indicating that each one-point reduction in global QoL score corresponds to a 2% increase in mortality risk.^42^ This finding suggests that HRQL assessments conducted pre- and post-treatment may serve as prognostic indicators. However, the meta-analysis incorporated only seven studies, and the subgroup analyses lacked robustness, necessitating cautious interpretation. Several other studies have demonstrated positive correlations between HRQL and overall survival, implying that interventions aimed at enhancing HRQL may also improve survival rates, both in patients with advanced disease and in those undergoing curative surgery.^43–47^

### Health-related quality of life

Although there is no universal consensus on the definition of HRQL, it is commonly viewed as it reflects a multidimensional concept that captures the physical, psychological, and social aspects that affect a person’s overall QoL.^48^^,^^49^ This is reflected in the concept of cancer survivorship, which refers to the health and well-being of a person with cancer from the time of diagnosis to the end of life. Measuring HRQL is essential because it addresses the comprehensive impact of health conditions and medical treatments on an individual’s overall well-being, extending beyond traditional clinical outcomes, such as mortality and symptom presence.^50^^,^^51^ The assessment of HRQL is generally conducted through self-evaluation using PROMs that assess general health, including physical, mental, and social well-being and activity limitations.^52^ This approach primarily seeks to thoroughly assess the health-related aspects following the impact of illnesses and treatments.^50^ Therefore, HRQL measurements essentially assess how patients perceive their overall health.^50^^,^^52^

Despite a notable increase in the number of publications over the past 20 years,^3^^,^^53^ research on HRQL in individuals with esophageal cancer remains relatively limited compared to areas such as treatment strategies, disease mechanisms, and clinical outcomes.^54^^,^^55^ Although a broad range of HRQL domains is covered, their depth is often limited. Instruments such as the European Organisation for Research and Treatment for Cancer (EORTC) QLQ-C30, with the esophago-gastric cancer module, QLQ-OG25, offer extensive coverage; however, detailed analysis of specific constructs, such as psychological distress or multidimensional pain, remains rare, especially when considering long-term effects. Most studies examining HRQL following curative treatment for gastrointestinal (GI) cancer have concentrated on patients with esophageal cancer. Of the 49 studies involving curatively treated patients, most focused on esophageal cancer (*N* = 40), while 9 addressed gastric cancer.^56^

### Health-related quality of life after treatment

#### Curative intended treatment

Patients with esophageal cancer often experience deterioration in HRQL postoperatively, with symptoms such as eating restrictions, dysphagia, and deteriorated global QoL.^56^ A recent systematic review and meta-analysis revealed that patients with esophageal cancer experienced variations in short-term HRQL during the initial 4 months, depending on whether they underwent definitive chemoradiotherapy (dCRT), nCRT, or surgery alone. Patients who underwent nCRT and surgery demonstrated better global QoL than those who underwent dCRT in the short term (first 4 months). Nevertheless, after a year, no notable differences in HRQL were observed between these treatment methods.^56^ Furthermore, this study showed that esophagectomy caused a clinically relevant decline in global QoL, a measure of overall health and quality of life combined, whereas the decline following gastrectomy was less significant and not considered clinically relevant. Nevertheless, both methods led to a significant and enduring decline in various clinically important HRQL functions and symptoms. A different systematic review that included meta-analyses^57^ examined HRQL after dCRT for esophageal cancer. The findings indicated that global QoL remained consistent over time and showed improvement after 36 months compared with the initial assessment. After 6 months of treatment, several tumor-specific symptoms, including dysphagia, dietary restrictions, and pain, showed improvement compared to the pre-treatment period. However, dyspnea deteriorated after the same duration.

The ROBOT trial was a single-centre, RCT that compared open transthoracic esophagectomy with robot-assisted minimally invasive thoracoscopic esophagectomy in patients with resectable esophageal cancer.^58^ The results showed that the global QoL scores 6 weeks after discharge were better for the group that underwent robot-assisted minimally invasive thoracoscopic esophagectomy. Additionally, no HRQL measurements were reported in the 5-year follow-up.^59^

The TIME trial was a multicenter, open-label, randomized controlled trial that compared minimally invasive esophagectomy with open esophagectomy in patients with resectable esophageal cancer.^60^ The results indicated that the physical component summary of the SF-36 questionnaire was significantly better in the minimally invasive esophagectomy group. This group also reported higher global QoL, less pain, and fewer issues with speaking than the open esophagectomy group. Additionally, no HRQL measurements were reported at the 3-year follow-up.^18^

The MIRO trial compared hybrid minimally invasive esophagectomy with open esophagectomy in the treatment of esophageal cancer.^61^ Some HRQL benefits of hybrid minimally invasive esophagectomy, particularly in social functioning and pain, persisted for up to 2 years post-surgery. However, at the 3-year mark, no significant differences in any measured HRQL outcomes were observed between the hybrid minimally invasive esophagectomy and open esophagectomy groups. Additionally, no HRQL measurements were reported in the 5-year follow-up.^62^

The Randomised Oesophagectomy: Minimally Invasive or Open(ROMIO) study is a pragmatic RCT that compared hybrid (minimally invasive) surgery with open surgery for the treatment of localized esophageal cancer.^63^ The results showed no significant difference in physical function (as measured by the QLQ-C30 physical function scale) between the hybrid and open surgery groups during the first 3 months after random allocation. Unlike the MIRO and TIME studies, which reported fewer major complications and pulmonary infections associated with minimally invasive techniques, the ROMIO study did not identify any such benefit.

The Minimally invasive versus Open esophagectomy for thoracic Esophageal cancer Trial (MONET) is a recently published (preprint) multicenter, open-label, randomized, controlled phase 3 trial, that compared thoracoscopic and open esophagectomy for esophageal cancer.^64^ The results demonstrate non-inferiority in terms of overall survival. The study found that thoracoscopic esophagectomy led to significantly less intraoperative blood loss, lower incidence of postoperative bleeding, and better preservation of postoperative respiratory function than open esophagectomy. Postoperative HRQL was assessed using the QLQ-C30 questionnaire. No significant differences were observed in the proportion of patients with worsening global QoL scores at both 1 and 3 months after surgery between the two groups. Additionally, the functional and symptom scales from the QLQ-C30 were compared, revealing no major differences except for pain. At 3 months postoperatively, the thoracoscopic esophagectomy group reported less pain than the open esophagectomy group (*P* = 0.071), although this difference was not statistically significant.

A recent systematic review and network meta-analysis evaluated four surgical techniques for esophageal cancer: open esophagectomy, hybrid esophagectomy, laparoscopic minimally invasive esophagectomy, and robot-assisted minimally invasive esophagectomy.^65^ Although the study did not report any HRQL outcomes, it emphasizes the need for well-designed randomized trials to fill this gap in the literature.

nCRT and neoadjuvant chemotherapy (nCT) are preoperative treatment approaches for locally advanced esophageal cancer.^66^ Analysis of the results from key trials, such as CROSS, OE02, MAGIC, and FNCLCC-FFCD, which have informed the current treatment protocols, revealed that HRQL outcomes were similar for both approaches. Nevertheless, nCRT was linked to an increase in respiratory symptoms, whereas patients who underwent nCT tended to have more GI problems. Typically, nCRT results in higher rates of pCR and R0 resection. Both nCRT and nCT have enhanced survival rates compared with surgery alone.^66^

Research examining the long-term effects of esophagectomy on patients’ HRQL has yielded mixed findings. Some studies have reported a spectrum of impairments, ranging from none to chronic issues, such as reflux, fatigue, dyspnea, and insomnia.^67^^,^^68^ While some studies suggest that approximately half of the patients who have undergone esophagectomy for cancer experience HRQL similar to that of the general population, other studies indicate that most HRQL dimensions remain affected over the long term when compared to healthy reference groups.^69^^,^^70^ Additionally, research has shown that esophagectomy can lead to GI symptoms and a decline in HRQL that may last for more than 15 years post-surgery^16^^,^^71–73^ and as long as 20 years.^74^ Considering the relatively high average age at which esophageal cancer is diagnosed, it is crucial to address geriatric issues such as functional decline, social isolation, and symptoms of depression in these patients. These impairments are frequently associated with an elevated risk of adverse health outcomes, including increased mortality rates, functional or cognitive decline, treatment-related complications, extended hospital stays, and diminished HRQL.^75^

#### Palliative treatment

Research on HRQL in the context of palliative treatment for esophageal cancer is limited. A recent systematic review identified a few studies focusing exclusively on this diagnosis, as it is frequently grouped with gastric or gastroesophageal cancer. Consequently, few studies have used esophageal-specific tools such as the QLQ-OES18 questionnaire, and none have focused exclusively on HRQL progression in esophageal cancer.^43^ The same review found that regarding pure palliative systemic therapy, evidence indicates that patients reported impaired global QoL before the start of treatment, with mean scores of 54.6 and 57.9 for first-line and beyond first-line treatment settings, respectively. *First-line treatment refers to initial systemic therapy administered with palliative intent for patients with advanced esophagogastric cancer prior to any subsequent lines of therapy.* The most common problems were reduced global QoL, abdominal pain, anxiety, appetite loss, and fatigue. During treatment, global QoL generally remained stable, with 81% of the treatment arms showing no significant changes. In the first-line setting, improvements were observed in various symptoms and emotional functioning, whereas in the beyond first-line setting, deterioration was noted in role functioning, fatigue, and distress related to hair loss. However, after disease progression, global QoL, cognitive and social functioning, and physical and role functioning can worsen.^76^

#### Immunotherapy

pCR of treatment is associated with both overall and recurrence-free survival.^77–79^ Given the poor prognosis of esophageal cancer, even in patients treated with curative intent, the impact of immunotherapy has become a prominent topic in esophageal cancer research. Checkpoint blockade has emerged as an effective treatment.^17^ A prior systematic review and meta-analysis indicated that the use of immune checkpoint inhibitors alongside chemotherapy or chemoradiotherapy as neoadjuvant treatment for locally advanced esophageal cancer notably improved pCR rates compared to standard neoadjuvant therapy.^80^ Additionally, one study found that patients receiving adjuvant nivolumab had significantly longer recurrence-free survival than those who did not.^81^ Immune checkpoint inhibitors have been associated with improved HRQL and a longer duration before clinical decline on several patient-reported outcome (PRO) scales compared with chemotherapy in a range of solid tumors.^82^ In the KEYNOTE-181 RCT, pembrolizumab demonstrated a significant survival advantage over chemotherapy in patients with metastatic squamous cell carcinoma of the esophagus and led to a lower incidence of grade 3–5 treatment-related adverse events. HRQL data, along with the survival benefit, support pembrolizumab as a second-line therapy for patients with advanced esophageal cancer.^83^ In the ORIENT-15 study, a phase 3 clinical trial with random assignment, researchers assessed the effectiveness of sintilimab combined with chemotherapy as an initial treatment for patients with advanced esophageal squamous cell carcinoma. The findings showed that those receiving the sintilimab and chemotherapy combination maintained or improved HRQL compared to patients who received chemotherapy only, with a median follow-up of 32.2 months. Significant changes were observed in various QLQ-C30 scales, such as the social functioning, pain, fatigue, constipation, and QLQ-OES18 scales, which evaluate pain, difficulty swallowing saliva, and choking while swallowing. The sintilimab group also demonstrated a reduced risk of deterioration in dysphagia and challenges with swallowing saliva. Furthermore, patients who exhibit superior HRQL performance across various scales experience improved overall survival.^84^ Some checkpoint inhibitors have also been shown to be more cost-effective than chemotherapy.^85^

### Interventions to improve health-related quality of life after treatment

Although interventions specifically designed to enhance HRQL following esophageal cancer treatment are limited, Enhanced Recovery After Surgery (ERAS) protocols present a promising approach. Initially developed to mitigate surgical stress and accelerate recovery, these protocols encompass a variety of evidence-based practices, including early mobilization, structured physical activity (prehabilitation), optimized nutritional strategies, and patient education. However, there is a significant paucity of RCTs conducted to date,^86^ leaving the benefits of ERAS in esophageal surgery open for further assessment. Self-management remains an essential component of care for survivors of cancer. A study by Schandl *et al*.^87^ underscores a substantial gap in evidence-based self-care guidance specifically tailored for esophageal cancer survivors. Most available advice is generally disseminated by fellow survivors rather than grounded in systematic research. Mobile application solutions for cancer management have proliferated rapidly, and the development of cancer-specific applications each year far exceeds empirical evidence supporting their effectiveness.^88^^,^^89^ This disparity has resulted in what is termed an ‘evaluation crisis’ in the field, as innovation has outpaced rigorous validation.^90^ The domains of exercise and nutritional support are the most extensively investigated areas in the context of interventions to improve HRQL in patients with esophageal cancer.

#### Exercise

Engaging in physical activity has been suggested as a possible way to improve HRQL, with several systematic reviews across different cancer types showing positive results.^91^^,^^92^ Nevertheless, a recent systematic review with meta-analysis^93^ revealed that physical exercise interventions post-surgery did not significantly affect HRQL compared to the control group pooled mean difference of 0.77 [95% CI −4.36, 5.90]. It is crucial to recognize that the quality of the evidence was very low, which should be considered when interpreting these findings. A systematic review and meta-analysis by Thomsen *et al*.^94^ suggested that there might be an increased risk of harm linked to exercise in cancer patients receiving systemic treatment. Nonetheless, the authors stressed that the evidence is still inconclusive and that there is a lack of sufficient data to thoroughly assess the risks and benefits of structured exercise in this group. Evidence regarding patients with esophageal cancer and postsurgical exercise remains inconclusive; therefore, no recommendations can be made either for or against.^93^ The same applies to overall evidence concerning prehabilitation and its impact on enhancing HRQL outcomes. The evidence is primarily constrained by the heterogeneity in intervention and outcome assessment, making it impossible to recommend either for or against prehabilitation, similar to the situation with post-surgery exercise.^95^^,^^96^ Subsequent to the most recent systematic review, the Pre-EMPT study was published to evaluate the effects of a structured prehabilitation exercise program on patients undergoing nCT and esophagectomy for esophageal adenocarcinoma.^97^ Prehabilitation has the potential to positively affect HRQL. After the nCT, the intervention group exhibited improvements in cognitive, emotional, and sleep function scores. Furthermore, prehabilitation may enhance the response to chemotherapy, as evidenced by the fact that the intervention group achieved superior chemotherapy response rates compared with the control group. Nonetheless, these findings should be approached with caution because of the small and non-randomized nature of the study. The CHALLENGE (Colon Health and Lifelong Exercise Change) Trial produced highly promising outcomes. Conducted across 55 centers, the trial included 889 patients with resected stage III or high-risk stage II colon cancer who had completed nCT. Participants were randomly assigned to either a structured exercise program or to receive only health education materials over a 3-year period. At a median follow-up of 7.9 years, the exercise group exhibited significantly longer disease-free survival than the health education group (hazard ratio, 0.72; 95% CI, 0.55−0.94; *P* = 0.02). The 5-year disease-free survival rates in the exercise and health education groups were 80% and 74%, respectively. Furthermore, the results indicated longer overall survival in the exercise group (hazard ratio for death, 0.63; 95% CI, 0.43 0.94). This landmark study provides robust evidence supporting the integration of structured exercise into the care regimen for colon cancer survivors. While the findings from the CHALLENGE and Pre-EMPT studies are encouraging, studies evaluating whether the results can be replicated in patients with esophageal cancer must be conducted before making any recommendations.

#### Diet

A systematic review and meta-analysis found that home enteral nutritional support is an effective way to support patients who have undergone treatment for upper GI cancer.^98^ This study demonstrated that this approach leads to a better global QoL than a regular oral diet. This may be due to the fact that many patients feel stressed about inadequate nutritional intake and associated weight loss. Another systematic review and meta-analysis investigated the effects of early oral feeding after esophagectomy.^99^ This study showed that early oral feeding significantly improved the global QoL of patients after esophagectomy. It also substantially reduces symptoms of dysphagia and difficulties with eating. However, early oral feeding did not significantly affect reflux or pain. A Cochrane systematic review examined the effects of preoperative nutritional therapy compared with usual care in patients undergoing gastrointestinal surgery.^100^ The authors concluded that they were unable to determine whether the different types of nutritional therapy had any effect on clinical outcomes due to very low certainty of evidence. A recent systematic review highlighted a significant gap in the current literature regarding exercise and nutritional interventions for patients with advanced esophageal cancer, as no studies were found.^101^ In a comprehensive review of broader evidence concerning diet and cancer, an analysis of 252 RCTs, including 31,067 patients, revealed limited evidence supporting dietary interventions as a therapeutic strategy in cancer treatment. Significantly, only 6% of these studies used standardized and validated QoL measurements as the primary endpoint.^102^

### Family caregivers of esophageal cancer patients

Research on family caregivers of patients with cancer has increased over the last two decades. Although research on family caregivers of patients with esophageal cancer remains limited, existing studies have consistently reported that caregivers experience considerable challenges in their daily lives. Patients’ HRQL is negatively affected by both diagnosis and treatment, and research suggests that this, in turn, affects the emotional functioning of family caregivers.^103^ Other factors influencing family caregivers’ HRQL include age, education level, and patient complications.^104^ Furthermore, high caregiver burden has been associated with poor health outcomes.^105^ In addition, approximately one-third of the family caregivers of patients with esophageal cancer who underwent treatment with curative intent experienced moderate to high levels of caregiver burden 3 years after treatment. The primary factors linked to caregiver burden at this 3-year mark were patient fatigue and caregiver depression.^106^ A study examining the role of benefit-finding as a mediator between caregiver burden and symptoms of anxiety and depression suggested that future research should concentrate on benefit-finding interventions to reduce such symptoms in family caregivers and enhance the quality of care for patients.^107^ Moreover, research suggests that positive focus coping strategies may play an important role in reducing psychological distress.^108^

Research has also explored other psychosocial elements such as the financial strain experienced by caregivers. This financial strain arises from time devoted to activities related to illness, leading to a decrease in productivity. This study revealed that the frequent requirement of a family caregiver adds an extra burden, affecting their ability to sustain employment and limit their personal lives.^109^

One study found that in the immediate period following hospital discharge, family caregivers felt abandoned and solely responsible for patients’ care at home, which is a life-changing situation.^110^ Two years after treatment, family caregivers experienced distress related to patients’ nutritional status, fear of recurrence, and anxiety about the future.^111^ Additionally, the psychosocial aspects of the transition from family members to caregivers have been highlighted.^104^^,^^111^

## RESULTS OF THE EVIDENCE AND GAP MAP

The full description of the included studies covered conflicts of interest, control group intervention, country, date of literature search, first author, meta-analysis (yes/no), number of studies included in the review (with HRQL outcome), outcomes (questionnaires and/or domains/study-specific questions), publication year, source of financial support, title, type of intervention, and timing of outcome assessments (Supplementary material 2).

### Scope of included evidence

From the database searches, 7420 references were identified. Duplicates were removed (n = 2369), leaving 5049 studies for screening. A total of 199 studies were included (the PRISMA flowchart and search strategy are provided in Supplementary material 1. Quality assessment of the included systematic reviews was performed using A MeaSurement Tool to Assess systematic Review 2 (AMSTAR-2)^112^ or the International Society for Pharmacoeconomics and Outcomes Research (ISPOR) checklist.^113^ Of the 24 reviews included, 19 (79%) were rated as critically low or insufficient quality, 2 (8%) as low quality, and 3 (13%) as high quality. A full assessment of the AMSTAR-2 and ISPOR quality ratings is presented in Supplementary material 1.

### Distribution across health-related quality of life domains and interventions

All the HRQL domains and interventions were coded into categories. The final results consisted of seven HRQL domains (overall QoL, physical functioning & performance, emotional & psychological health, social functioning & relationships, symptom burden & disease-specific symptoms, role functioning & daily activities, cognitive & existential well-being) and seven interventions (surgical interventions, chemotherapy/chemoradiotherapy, stenting and palliative surgical therapies, nutritional interventions, supportive care and lifestyle interventions, immunotherapy, traditional and complementary medicine). Supplementary material 3 presents a frequency table of the extent to which specific HRQL measures or items were applied within each type of intervention. Supplementary material 1 shows the distribution of all questionnaires and the individual items used in the included studies.

### Key patterns and gaps identified

**Figure 1:**
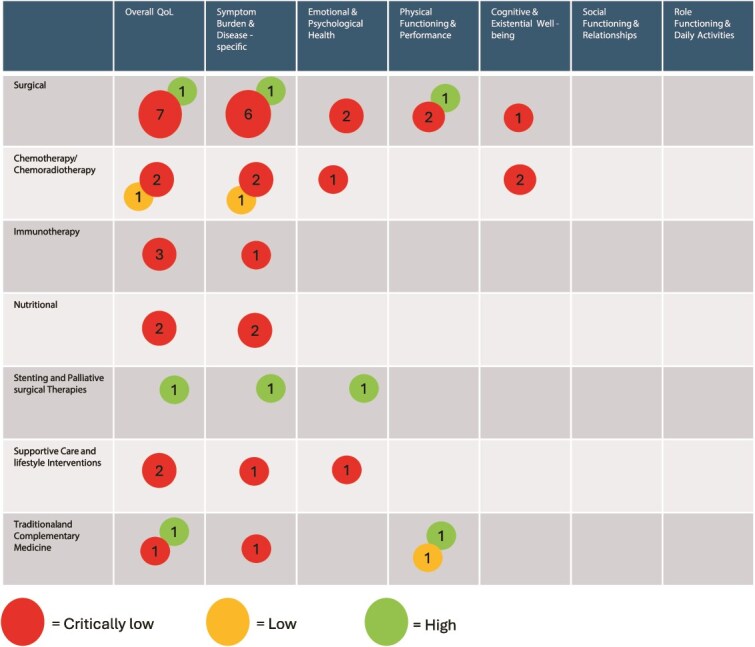
Mapping Interventions for health-related quality of life domains. This figure illustrates interventions (rows) mapped against HRQL domains (columns), with each cell representing the number and quality of systematic reviews identified. Each systematic review was counted only once per HRQL domain, even if it included multiple HRQL instruments addressing the same domain. In total, 84 unique HRQL questionnaires/items were extracted, but overlapping instruments for a specific HRQL domain within a single review were not double-counted. For example, if one review included both QLQ C-30 and EQ-5D as outcomes, it contributed a count of 1 to the “Global Health & Overall QoL“ domain. The size of the circles corresponds to the number of systematic reviews covering that domain. The colour of the circles represents the highest AMSTAR-2/ISPOR rating among reviews within that cell (high, moderate [no review was rated moderate], low, or critically low confidence). Numbers inside the circles indicate the count of systematic reviews. Empty cells denote areas with no systematic reviews of any AMSTAR-2 confidence level, indicating evidence gaps.

**Figure 2:**
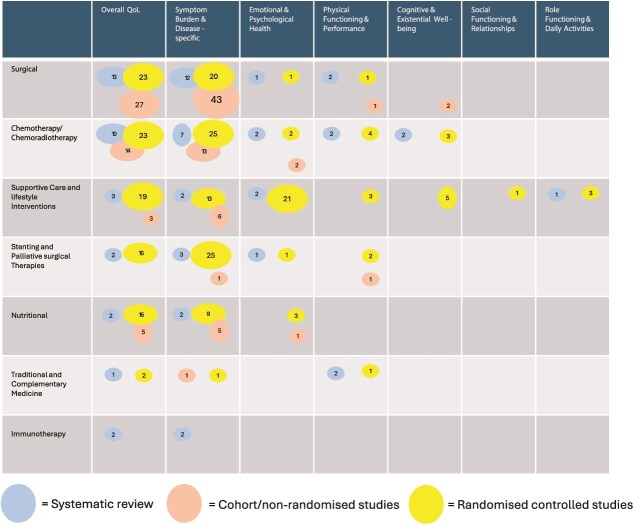
Mapping Interventions for health-related quality of life domains. The figure illustrates interventions (rows) mapped against HRQL domains (columns), with each cell representing the number of studies, categorised by study design (systematic reviews, randomised controlled trials, and cohort/non-randomised studies). In total, 84 unique HRQL questionnaires/items were extracted, and each study was counted as many times as the number of HRQL questionnaires/items it reported in a given domain. For instance, if a study assessed overall QoL using both the QLQ-C30 and EQ-5D, it contributed two counts to that domain. The size of the circles reflects the total number of studies within each cell. The colour of the circles indicates the type of study design. Numbers inside the circles show the count of studies contributing to the total. Empty cells reflect the absence of that study type for the given domain. Together, the maps highlight both areas of research concentration, as well as gaps where future research could be directed. Six outcomes were not specified or had a research specific questionnaire.

This EGM, which includes 84 distinct HRQL instruments, highlights both synthesis and absolute evidence gaps across HRQL domains. Most evidence originates from RCTs and cohort/non-randomized studies, with systematic reviews being less common. Absolute gaps refer to areas lacking identified studies, while synthesis gaps pertain to areas with poor-quality evidence. The synthesis map (Fig. 1) indicates that no HRQL domain was backed by a high-confidence body of systematic reviews, with most reviews rated critically low or low. The most frequently studied interventions were surgical and chemotherapy/chemoradiotherapy, which were primarily evaluated for overall QoL and symptom burden. The absolute gap map (Fig. 2) revealed that most studies focused on surgery, chemotherapy/chemoradiotherapy, supportive care, and lifestyle interventions, mainly assessed using overall QoL, symptom burden, and emotional and psychological health. Several areas remain unexplored, including stenting and palliative and surgical therapies; nutritional interventions; traditional and complementary medicine; immunotherapy; and evaluation of cognitive and existential well-being, social functioning, relationships, as well as role functioning and daily activities.

### Limitations

A notable limitation of this EGM is the classification of the HRQL domains and interventions. Numerous HRQL instruments, particularly those that are widely utilized, such as the QLQ-C30, encompass multiple domains including cognitive, social, and role functioning. To enhance mapping and clarity, each questionnaire was assigned a primary domain, based on its principal focus. For example, the QLQ-C30 was categorized under ‘Overall Quality of Life’ despite having several subscales pertinent to other domains. Consequently, certain areas may appear as complete evidence gaps (e.g. cognitive or social functioning), even though they are partially addressed within broader instruments. This simplification may lead to an underrepresentation of evidence in specific domains, but it was deemed necessary to present a structured and interpretable overview of the literature. Similarly, intervention categories were broadly grouped to capture general trends, which might obscure nuances in treatment approaches or mixed intervention designs (e.g. multidisciplinary programs involving both physical and psychological components). Owing to time constraints and the broad scope of this Evidence and Gap Map, we did not conduct formal risk of bias assessments for individual primary studies using tools such as ROB 2 or ROBINS-I. This decision aligns with Campbell Collaboration guidance, which recommends, but does not require, such appraisal for primary studies in EGMs. Instead, we prioritized the critical appraisal of systematic reviews and employed rapid methods throughout, as outlined in the methodology. We acknowledge that the absence of detailed internal validity assessments for primary studies may limit the interpretive depth regarding the reliability of individual trial findings. Despite these limitations, the EGM effectively provides a comprehensive view of scientific evidence on HRQL and interventions for esophageal cancer. It highlights clear gaps in both primary research and evidence synthesis and identifies domains and intervention types where future research efforts should be prioritized.

## DISCUSSION

### Reporting bias in studies with health-related quality of life outcomes

A large study conducted a meta-analysis of PROMs in RCT protocols and their subsequent reporting in trial publications.^114^ This study analyzed 488 RCTs. The findings revealed that 30% of the examined RCT protocols did not include PROMs, while 20% used PROMs as primary outcomes and 50% as secondary outcomes. Eight to ten years after the approval of the RCTs, the results were available for 77% of the trial protocols that had prespecified PROMs. Nevertheless, only 44% of the published trials included all PROs as outlined in the protocol. Furthermore, 21% failed to report any of the prespecified PROMs, and 36% presented PROMs that were more, fewer, or different from those initially specified in the protocol. These results indicate large issues with outcome reporting bias. A comparable study was conducted in a cancer context, encountering similar issues with a reporting bias. Between 2000 and 2017, 76 phase III trials focusing on therapeutic interventions for GI cancer were registered at ClinicalTrials.gov. In most of these studies (86%), the focus was on disease-related outcomes such as progression-free or overall survival. Although every trial included secondary endpoints for adverse events, only 39% incorporated QoL measures as secondary endpoints. These included studies on upper GI cancer (n = 21), pancreatic cancer (n = 12), hepatobiliary cancer (n = 11), and colorectal cancer (n = 32). Upper GI cancer included patients with either gastric or esophageal cancer. Among the 21 upper GI studies, 11 trials considered HRQL as a secondary outcome measure; however, only three studies published HRQL results. Notably, none of the supporting trials designated HRQL as the primary endpoint.^115^

### Assessment of health-related quality of life

Numerous PROMs have been used to assess HRQL in patients with esophageal cancer. Among these, the QLQ-C30^116^ is the most frequently used.^56^ It evaluates the overall quality of life through five functional domains—physical, role, emotional, cognitive, and social— along with three symptom scales, a global health status scale, and additional items. Its strengths are attributed to its robust content validity and scale structure^117^^,^^118^ while showing limited or mixed evidence for properties such as internal consistency, test–retest reliability, and responsiveness.^117^ For esophageal cancer, the QLQ-C30 is frequently combined with disease-specific modules such as the QLQ-OES18, QLQ-OES24, and more recently, the QLQ-OG25.^119^ The QLQ-OES24, developed by Blazeby *et al*.,^120^ consists of 24 items that address dysphagia, reflux, pain, eating difficulties, and other upper gastrointestinal symptoms. The QLQ-OES24 was later revised to improve psychometric properties, clinical relevance, and patient acceptability, as well as to increase sensitivity to treatment-related changes in QoL; therefore, the scale was reduced to 18 items.^121^ Recent evidence has shown that it can reliably and validly measure symptom severity in patients with advanced or metastatic esophageal squamous cell carcinoma.^122^ The QLQ-OG25, designed to cover both esophageal and gastric cancers for symptom-specific problems, has shown acceptable reliability and validity.^119^^,^^123^ However, to date, only a limited number of systematic reviews have comprehensively evaluated HRQL instruments in cancer populations while fully adhering to the guidelines established by the Consensus-based Standards for the Selection of Health Measurement Instruments (COSMIN).^124^ These guidelines are considered the gold standard for assessing the psychometric quality of PROMs. Many previous reviews have not clearly presented their rating results, assessed the risk of bias, or effectively graded the quality of the evidence. An upcoming systematic review following the COSMIN methodology aims to fill these gaps by providing a comprehensive evaluation of PROMs used to assess HRQL throughout the cancer continuum in Europe.^125^ The findings from this review are expected to inform future research and assist in clinical decision-making regarding the selection of instruments.

Although there are specific PROMs designed for various esophageal conditions, a universal PROM that evaluates both malignant and benign esophageal diseases has not been previously reported. To fill this gap, the Cleveland Clinic Esophageal Questionnaire (CEQ) was developed with the explicit aim of serving as a universal PROM applicable to all esophageal diagnoses.^126^ The CEQ comprises 34 items across six domains: dysphagia, eating, pain, reflux and regurgitation, dyspepsia, and dumping syndrome. It also includes a question assessing the ‘bother’ experienced by patients, which evaluates its impact on QoL. One limitation of the CEQ is that it is organ-specific and does not address broader areas such as psychological well-being.

Nevertheless, in trials where a measurable therapeutic effect has been observed, disease-specific instruments show significantly greater responsiveness (mean = 0.57) than generic instruments (mean = 0.39, *P* = 0.01) and their related domains (mean = 0.40, *P* = 0.03).^127^ While utilizing a single PROM may simplify administration, it may also lead to reduced sensitivity to changes specific to the condition being assessed.^127^ A systematic review and meta-analysis found that the overall prevalence of dumping syndrome after surgery for esophageal cancer was 27% (95% CI: 14%–39%). The prevalence reported in individual studies ranges from 0% to 74%. However, subgroup analyses revealed that studies employing specialized questionnaires yielded a higher pooled prevalence of 67% (95% CI: 60%–73%) with less variability. This study highlights the importance of using PRO questionnaires for accurate assessments.^128^ A balanced approach could involve combining general PROM with a disease-specific approach, allowing researchers and clinicians to capture both broad and condition-specific outcomes.^129^

A Delphi study was conducted to establish standardized definitions, investigations, and management strategies for GI symptoms and conditions following esophagogastric cancer surgery.^130^ This study employed a modified two-round Delphi consensus method involving a multidisciplinary panel of experts to achieve a consensus on these matters. The study successfully reached an agreement on defining 26 symptoms and 10 conditions commonly experienced by patients after esophagogastric cancer surgery. Of the 26 symptoms, 13 were rated as important, whereas 8 of the 10 conditions were rated as important. Nonetheless, other evidence suggests a divergence between the perceptions of critical symptoms held by healthcare professionals and patients. Clinicians predominantly focus on upper GI symptoms, whereas patients tend to report a broader range of issues, including lower GI symptoms.^131^

A core outcome set has been established for efficacy trials of esophageal cancer resection surgery.^132^ This set recommends the assessment of several key parameters, including overall survival, mortality during hospitalization, surgical inoperability, requirement for additional surgical procedures, pulmonary complications, necrosis of the conduit and anastomotic leakage, severe nutritional issues, capacity for oral consumption, symptoms of acid reflux or heartburn, and overall QoL. Concurrently, efforts are underway to develop a separate core outcome set for exercise interventions for esophagogastric cancer^133^

### Implications and recommendations from the evidence and gap map

Researchers can use the EGM and other established priorities in survivorship research ^134^ as strategic tools to effectively prioritize and design future studies. By identifying both synthesis gaps and absolute evidence gaps, this map offers a visual and analytical foundation for directing research efforts toward areas of greatest need and potential impact. For instance, the lack of robust evidence on cognitive and existential outcomes, social functioning, and the effects of nutritional or immunotherapy interventions on HRQL highlights concrete opportunities for targeted investigation. Furthermore, researchers could use theoretical models to guide interventions for managing long-term physical effects and prioritize population-wide monitoring of psychosocial outcomes, as well as the development and evaluation of psychosocial interventions.^134^ Researchers planning trials, systematic reviews, or other types of studies can leverage the map to avoid redundancy, ensure alignment with patient-relevant outcomes, and optimize the value of their work. Consequently, the EGM serves not only as a summary of current knowledge but also as a roadmap for a more patient-centered, evidence-informed research agenda in esophageal cancer care. The EGM highlights several critical areas for future research. First, high-quality longitudinal studies that assess HRQL beyond the immediate postoperative period are needed, particularly among long-term survivors. The cognitive, social, and existential domains remain underexplored, despite their potential impact on survivorship. Second, future trials should prioritize the inclusion of HRQL as a primary or key secondary endpoint, utilizing both general and disease-specific PROMs. The underreporting of HRQL outcomes underscores the need for standardized reporting frameworks and adherence to CONSORT-PRO guidelines.^135^ Finally, efforts in evidence synthesis should aim to improve the methodological quality of the systematic reviews. The core outcome sets for HRQL in esophageal cancer were adopted to ensure comparability across studies. Additionally, earlier in the text, we described an upcoming comprehensive evaluation of PROMs used to assess HRQL throughout the cancer continuum in Europe.^125^ In parallel, a scoping review by Crump *et al*. will specifically assess the psychometric quality of PROMs for esophageal cancer using the COSMIN standards.^136^ Together, these complementary resources can guide researchers in identifying outcomes that require further investigation and in determining PROMs that are sufficiently psychometrically robust for future studies.

## Conflicts of interest

The authors declare no conflicts of interest.

## Supplementary Material

Supplementary_material_1_doaf086

Supplementary_material_2_study_charachteristics_doaf086

Supplementary_material_3_Outcomes_and_interventions_doaf086

## Notes

Specific author contributions: Kenneth Färnqvist (Conceptualization, Data curation, Formal analysis, Investigation, Methodology, Project administration, Visualization, Writing—original draft, Writing—review & editing), Kalle Mälberg (Formal analysis, Investigation, Writing—review & editing), Sophie I. Johnsson (Conceptualization, Writing—review & editing), Asif Johar (Conceptualization, Supervision, Writing—review & editing), Anna Regina Schandl (Conceptualization, Supervision, Writing—review & editing), Cecilia Ringborg (Formal analysis, Investigation, Writing—review & editing), and Pernilla Lagergren (Conceptualization, Funding acquisition, Project administration, Supervision, Writing—original draft, Writing—review & editing)
