# Correction to: Health-related quality of life in esophageal cancer: a state-of-the-art review of patient-reported outcomes and an evidence and gap map

**DOI:** 10.1093/dote/doaf097

**Published:** 2025-10-28

This is a **correction** to:

Kenneth Färnqvist, Kalle Mälberg, Sophie I Johnsson, Asif Johar, Anna Schandl, Cecilia Ringborg, Pernilla Lagergren, Health-related quality of life in esophageal cancer: a state-of-the-art review of patient-reported outcomes and an evidence and gap map, *Diseases of the Esophagus*, Volume 38, Issue 5, October 2025, doaf086, https://doi.org/10.1093/dote/doaf086

In the originally published version of this manuscript, there is an incorrect statement in the Clinical Context section. This sentence reads, ``Esophageal cancer is a significant global health issue, with approximately 1.6 million cases reported in 2020.'' This is incorrect; the number should be 600,000. Therefore, the sentence should read: ``Esophageal cancer is a significant global health issue, with approximately 600,000 cases reported in 2020.''

This error has been corrected.

