# Supplementary material for: Health-related quality of life in esophageal cancer: a state-of-the-art review of patient-reported outcomes and an evidence and gap map
Source: Dis Esophagus. 2025 Oct 12;38(5):doaf086. doi: 10.1093/dote/doaf086 (PMC12515475; doi:10.1093/dote/doaf086)
Supplement: Supplementary_material_1_doaf086 [file supplementary_material_1_doaf086.docx]

**Methods**

This study adhered to the Campbell Collaboration guidelines for developing this Evidence and Gap Map (EGM) (1). The process encompassed formulating a research question, establishing inclusion and exclusion criteria, conducting a systematic literature search, extracting and analysing the data, and visualising the results. Further, the methodology has 'rapid' methodological approaches at particular stages, influenced by the recommendations provided by the Cochrane Rapid Reviews Methods group (2).

**Eligibility**

Studies that met the eligibility criteria outlined in Table 1 were included in the meta-analysis.

**Table 1. Eligibility criteria.**

|  | **Inclusion** | **Exclusion** |
| --- | --- | --- |
| **Population** | Adults, age 18 or above, with oesophageal cancer  at any stage. Studies that include populations with broader criteria were included if the population mean or median age is above 18. | Studies including individuals with other types of cancer.  Animal studies. |
| **Intervention of interest** | Any type of intervention delivered to adults with oesophageal cancer. |  |
| **Comparator** | Any type of comparator. |  |
| **Outcome** | Any type of HRQL. | Studies only  focusing on oesophageal cancer diagnosis, assessment, or prevalence. |
| **Setting** | Any setting. |  |
| **Study design** | Systematic reviews, including at least three studies. Was expanded to RCTs, and interventional non-randomised studies or cohort studies where the exposure was interventional (e.g open surgery versus minimally invasive) if fewer than 50 reviews were found. | Cross-sectional studies, case studies, case series, conference abstracts, grey literature, cohort or case-control studies without two different interventions, and protocols, as well as scoping reviews. |
| **Publication types** | Peer-reviewed studies published in indexed scientific databases. |  |
| **Language** | English |  |
| **Publication or Report Date** | Publication/report date as of January 1, 2000, and onwards. |  |

**Search Strategy**

The search strategy was developed in Medline (Ovid), in collaboration with librarians at the Karolinska Institute University Library. Medical Subject Headings (MeSH) terms and free text terms were identified for each search concept. The search was translated into other databases using Polyglot Search Translator (3). Databases were searched from January 1 2000 onwards. Language restrictions were set in English. Limiting a systematic review to include only publications in English does not seem to alter the conclusions of such reviews (4).

The strategies are peer-reviewed by another librarian before execution. Deduplication was performed as previously described by Bramer et al. (5). We searched the general databases MEDLINE and Embase for relevant literature. The initial inclusion criteria were only systematic reviews that examined the impact of interventions on health-related quality of life (HRQL) in oesophageal cancer. If fewer than 50 systematic reviews meeting the specified inclusion criteria were identified, or if key interventions remained insufficiently addressed, the inclusion criteria were expanded to incorporate relevant primary studies (i.e. randomised controlled trials [RCTs] and interventional cohort studies). Fewer than 50 systematic reviews were eligible for inclusion; therefore, RCTs and cohort studies were included.

**Study selection**

Records identified from the search were imported into Covidence. An additional round of duplicate checks was conducted within Covidence. Screening forms for abstracts and full texts were developed and piloted for calibration (by two reviewers). Dual screening was performed in 10% of the records. If an acceptable level of agreement (κ ≥ 0.8) was achieved, then a single screening approach was implemented. The agreement was met, and a single-screen approach was implemented. In cases where a single screener was uncertain about inclusion, it was resolved through discussion with a team member.

**Data extraction**

A data extraction form (Excel sheet) was developed and piloted using a sample from two studies for calibration. The form captured conflicts of interest, control groups, country, date of literature search, first author, meta-analysis (yes/no), number of studies included in the review, outcomes (questionnaires and domains), population, publication year, setting, source of financial support, title, type of intervention, and timing of outcome assessment. All the outcomes and interventions were coded into categories. If new subcategories arose during the extraction phase, they were discussed with the research group, which could lead to new subcategories (interventions and outcome domains). Studies included in the systematic review were reviewed to determine the coding for the systematic review. One reviewer extracted the outcome data and study characteristics. If needed, a second team member was contacted to clarify uncertainties.

**Data cleaning**

One team member (not the extractor) examined a random sample (10% of the sample) of coded records to check for any code which appeared to be incorrectly used by the coder.

**Risk of bias**

A single reviewer assessed each identified systematic review using the AMSTAR-2 or ISPOR (6, 7) tool to evaluate the overall confidence in the review results. Uncertainties were resolved by consultation with a second reviewer. An overall judgement of quality was assigned to each included systematic review as a reflective measure of the strength of confidence in the results obtained from the review (quality), with four levels of quality: high, moderate, low, or critically low. The author's team made all of the consensus judgments. If systematic reviews included studies other than RCTs, we followed the method of Ringsten et al. for AMSTAR-2 assessments (8).

**Synthesis and presentation of findings**

The overall study characteristics were presented in a tabular format. Furthermore, the results were presented visually to provide a more comprehensive representation, facilitating a clearer understanding of the state of evidence and identifying potential gaps (areas with limited or no evidence) and synthesis gaps (areas with poor-quality evidence). The timing of the outcomes was planned to be categorised into the following groups: short-term (0-6 months), medium-term (>6-12 months), and long-term (>12 months). However, many studies did not report the timing of the outcomes, and the timing can only be seen for each individual study (Appendix 2). As part of the AMSTAR-2/ISPOR assessments, data on conflicts of interest and funding sources were extracted; however, these two variables were not presented in any other manner.

**PRISMA flow chart for screening of studies**

References from databases/registers **(n = 7420)** (as **n = 7418** studies)

Embase (n = 4116)

MEDLINE (n = 3304)

**Identification**

Studies included in review **(n = 199)**

Studies excluded **(n = 4611)**

Studies not retrieved **(n = 0)**

Studies assessed for eligibility **(n = 438)**

Studies sought for retrieval **(n = 438)**

Studies screened **(n = 5049)**

References removed **(n = 2369)**

Duplicates identified manually (n = 5)

Duplicates identified by Covidence (n = 2364)

Marked as ineligible by automation tools (n = 0)

Other reasons (n = )

**Screening**

Studies excluded **(n = 239)**

Not in English (n = 1)

Wrong outcomes (n = 21)

Wrong comparator (n = 2)

Wrong indication (n = 3)

Retracted article (n = 2)

Wrong intervention (n = 1)

Wrong study design (n = 116)

Full-text not retrieved (n = 6)

Wrong patient population (n = 63)

Not 3 studies included in review (n = 24)

| Study | Q1 | ***Q2*** | Q3 | ***Q4*** | Q5 | Q6 | ***Q7*** | Q8 | | ***Q9*** | Q10 | ***Q11*** | Q12 | ***Q13*** | Q14 | ***Q15*** | Q16 | Overall quality |
| --- | --- | --- | --- | --- | --- | --- | --- | --- | --- | --- | --- | --- | --- | --- | --- | --- | --- | --- |
| Janmaat 2017 |  |  |  |  |  |  |  | |  |  |  | NM | NM |  |  | NM |  | High |
| Li 2018 |  |  |  |  |  |  |  | |  |  |  |  |  |  |  |  |  | Critically low |
| Al Yazeedi 2024 |  |  |  |  |  |  |  | |  |  |  | NM | NM |  |  | NM |  | Critically low |
| Wei 2007 |  |  |  |  |  |  |  | |  |  |  |  |  |  |  |  |  | High |
| Parameswaran 2008 |  |  |  |  |  |  |  | |  |  |  | NM | NM |  |  | NM |  | Critically low |
| Al-Batran 2010 |  |  |  |  |  |  |  | |  |  |  | NM | NM |  |  | NM |  | Critically low |
| Scarpa 2011 |  |  |  |  |  |  |  | |  |  |  |  |  |  |  |  |  | Critically low |
| Amdal 2013 |  |  |  |  |  |  |  | |  |  |  | NM | NM |  |  | NM |  | Low |
| Dai 2014 |  |  |  |  |  |  |  | |  |  |  |  |  |  |  |  |  | High |
| Liu 2017 |  |  |  |  |  |  |  | |  |  |  |  |  |  |  |  |  | Critically low |
| Kauppila 2017 |  |  |  |  |  |  |  | |  |  |  |  |  |  |  |  |  | Critically low |
| Taioli 2017 |  |  |  |  |  |  |  | |  |  |  | NM | NM |  |  | NM |  | Critically low |
| Bolger 2019 |  |  |  |  |  |  |  | |  |  |  | NM | NM |  |  | NM |  | Critically low |
| Zhou 2021 |  |  |  |  |  |  |  | |  |  |  |  |  |  |  |  |  | Insufficient, based on the ISPOR checklist |
| Tukanova 2022 |  |  |  |  |  |  |  | |  |  |  |  |  |  |  |  |  | Critically low |
| vandenBoorn 2020 |  |  |  |  |  |  |  | |  |  |  |  |  |  |  |  |  | Critically low |
| Zhang 2022 |  |  |  |  |  |  |  | |  |  |  |  |  |  |  |  |  | Critically low |
| Gupta 2024 |  |  |  |  |  |  |  | |  |  |  |  |  |  |  |  |  | Critically low |
| Walmsley 2023 |  |  |  |  |  |  |  | |  |  |  | NM | NM |  |  | NM |  | Critically low |
| Sun 2023 |  |  |  |  |  |  |  | |  |  |  |  |  |  |  |  |  | Insufficient, based on the ISPOR checklist |
| Pan 2024 |  |  |  |  |  |  |  | |  |  |  |  |  |  |  |  |  | Low |
| Su 2024 |  |  |  |  |  |  |  | |  |  |  |  |  |  |  |  |  | Critically low |
| Fontes 2024 |  |  |  |  |  |  |  | |  |  |  | NM | NM |  |  | NM |  | Critically low |
| Shi 2025 |  |  |  |  |  |  |  | |  |  |  |  |  |  |  |  |  | Critically low |

**Quality assessment of the included systematic reviews was performed using the AMSTAR-2 or ISPOR checklists.**

AMSTAR-2 items:

Q1: Did the research questions and inclusion criteria for the review include the components of population, intervention, comparison, outcome (PICO)? Q2: Did the report of the review contain an explicit statement that the review methods were established prior to the conduct of the review and did the report justify any significant deviations from the protocol? Q3: Did the review authors explain their selection of the study designs for inclusion in the review? Q4: Did the review authors use a comprehensive literature search strategy? Q5: Did the review authors perform study selection in duplicate? Q6: Did the review authors perform data extraction in duplicate? Q7: Did the review authors provide a list of excluded studies and justify the exclusions? Q8: Did the review authors describe the included studies in adequate detail? Q9: Did the review authors use a satisfactory technique for assessing the risk of bias (RoB) in individual studies that were included in the review? Q10: Did the review authors report on the sources of funding for the studies included in the review? Q11: If meta-analysis was performed did the review authors use appropriate methods for statistical combination of results? Q12: If meta-analysis was performed, did the review authors assess the potential impact of RoB in individual studies on the results of the meta-analysis or other evidence synthesis? Q13: Did the review authors account for RoB in individual studies when interpreting/discussing the results of the review? Q14: Did the review authors provide a satisfactory explanation for, and discussion of, any heterogeneity observed in the results of the review? Q15: If they performed quantitative synthesis did the review authors carry out an adequate investigation of publication bias (small study bias) and discuss its likely impact on the results of the review? Q16: Did the review authors report any potential sources of conflict of interest, including any funding they received for conducting the review?

Items 2, 4, 7, 9, 11, 13 and 15 are AMSTAR 2 critical domains

Zhou (2021) and Sund (2023) were systematic reviews with network analyses and were therefore assessed using the ISPOR checklist (individual item assessments not shown). We converted the insufficient rating into a critically low rating to fit the AMSTAR 2 assessment when placing the ratings into the context.

NM= No meta-analysis

**Abbreviations for all included scales.**

1. **QLQ-C30**: Quality of Life Questionnaire-Core 30
2. **QLQ-OG25**: Quality of Life Questionnaire-Oesophago-Gastric 25
3. **QLQ-OES18/23/24**: Quality of Life Questionnaire-Oesophageal 18/23/24
4. **FACT-E**: Functional Assessment of Cancer Therapy-Oesophageal
5. **FACT-G**: Functional Assessment of Cancer Therapy-General
6. **FACT-HN**: Functional Assessment of Cancer Therapy-Head and Neck
7. **M-FACT-HN**: Modified Functional Assessment of Cancer Therapy-Head and Neck
8. **VAS (pain)**: Visual Analogue Scale (pain)
9. **FPS (Pain)**: Faces Pain Scale (Revised)
10. **SF-8/20/36**: Short Form Health Survey-8/20/36
11. **SDS**: Self-Rating Depression Scale
12. **PG-SGA**: Patient-Generated Subjective Global Assessment
13. **SAS**: Self-Rating Anxiety Scale
14. **HADS**: Hospital Anxiety and Depression Scale
15. **KPS**: Karnofsky Performance Status
16. **EPQ-RSC**: Eysenck Personality Questionnaire-Revised, Short Scale for Chinese
17. **ESCAS**: Exercise of Self-Care Agency Scale
18. **EQ-5D**: EuroQol-5 Dimensions
19. **M-RSCL**: Modified Rotterdam Symptom Checklist
20. **SQLI**: Spitzer Quality of Life Index
21. **DAUGS32**: Dysfunction After Upper Gastrointestinal Surgery for Cancer (32 items)
22. **DSQ**: DeMeester Symptom Questionnaire
23. **RSCL**: Rotterdam Symptom Checklist
24. **FACT**: Functional Assessment of Cancer Therapy
25. **POMS**: Profile of Mood States
26. **GERD-HRQL**: Gastroesophageal Reflux Disease Health-Related Quality of Life
27. **GSRS**: Gastrointestinal Symptom Rating Scale
28. **JSGS-Q**: Japanese Society of Gastroenterological Surgery Questionnaire for Dumping After Gastrectomy
29. **QOL-ACD**: Quality of Life Questionnaire for Cancer Patients Treated with Anti-Cancer Drugs
30. **ECOG**: Eastern Cooperative Oncology Group Performance Status
31. **CTCAE**: Common Terminology Criteria for Adverse Events
32. **RQS**: Reflux-Qual Short Form
33. **MRC-SI**: Medical Research Council Symptom Index
34. **ESAS**: Edmonton Symptom Assessment Scale
35. **SCL-90**: Symptom Checklist-90
36. **MFI**: Multidimensional Fatigue Inventory
37. **LADL**: Lawton Instrumental Activities of Daily Living Scale
38. **SMHSQ**: St. Mary’s Hospital Sleep Questionnaire
39. **PDI**: Patient Dignity Inventory
40. **PSQI**: Pittsburgh Sleep Quality Index
41. **GWB**: General Well-Being Scale
42. **WHO-PRS**: World Health Organization Psychosocial Response Scale
43. **Visick**: Visick Classification
44. **LASER**: Lasting Symptoms Over the Past 6 Months Questionnaire
45. **GERD-Q**: Gastroesophageal Reflux Disease Questionnaire
46. **HAM-A**: Hamilton Anxiety Rating Scale
47. **HAM-D**: Hamilton Depression Scale
48. **SMES**: Self-Management Efficacy Scale
49. **PFS-R**: Piper Fatigue Scale-Revised
50. **RDQ**: Reflux Disease Questionnaire
51. **SMCD**: Self-Efficacy for Managing Chronic Disease
52. **NHP**: Nottingham Health Profile
53. **PGSAS-45**: Post-Gastrectomy Syndrome Assessment Scale-45
54. **WEMWBS**: Warwick-Edinburgh Mental Well-Being Scale
55. **QLQ-INFO25:** Information module 25
56. **NCCN-DT:** National Comprehensive Cancer Network-recommended Distress Thermometer
57. **DRI:** Disability rating index
58. **PAIS**: Psychosocial Adjustment to Illness Scale after surgery
59. **PHQ**-9: Patient Health Questionnaire-9
60. **BPI**: Brief Pain Inventory
61. **BAI**: Beck Anxiety Inventory
62. **BDI-II**: Beck Depression Inventory-II
63. **QOLS:** The Quality of Life Scale
64. **CD-RISC:** Davidson Resilience Scale
65. **SUPPH:** Strategies used by people to promote health
66. **MCMQ:** Medical Coping Modes Questionnaire
67. **PSSS:** Perceived Social Support Scale
68. **WHOQOL:** World Health Organization Quality of Life questionnaire
69. **QLQ-STO22:** Stomach Cancer Module 22
70. **CTAE:** Common Terminology Criteria for Adverse Events (cough)
71. **LIADL:** Lawton Instrumental Activities of Daily Living

| **Outcome Category** | **Included Concepts / Subscales** |
| --- | --- |

| 1. Overall QoL | QLQ-C30, EQ-5D, SF-8/20/36, SQLI, QOL-ACD, WHOQOL, NHP, GWB, QOLS,VAS (self-rated health), QOL score (A lot better, A little better, The same, A little worse, A lot worse), QOL scale (12 grades, 60 points) |
| --- | --- |

| 2. Physical Functioning & Performance | KPS, ECOG, MFI, DRI, LADL, CTCAE, SMCD, PFS-R |
| --- | --- |

| 3. Emotional & Psychological Health | HADS, SAS, SDS, HAM-A, HAM-D, POMS, BDI-II, BAI, SCL-90, PHQ-9, EPQ-RSC, WEMWBS, WHO-PRS, CD-RISC, NCCN-DT, MCMQ |
| --- | --- |

| 4. Social Functioning & Relationships | PSSS, PAIS |
| --- | --- |

| 5. Symptom Burden & Disease-specific | QLQ-OG25, QLQ-OES18/23/24, FACT-E, DSQ, PG-SGA, GERD-HRQL, GSRS, DAUGS32, RQS, LASER, GERD-Q, RDQ, GS-Q, M-RSCL, RSCL, ESAS, BPI, VAS (pain), Visick, PGSAS-45, MRC-SI, QLQ-STO22, CTAE (cough), FPS, Dysphagia %, Dumping %, Regurgitation %, Dysphagia (0-4 scale), VAS dysphagia, Odynophagia (pain on swallowing, Mild pain, Moderate pain, Severe pain), Regurgitation (None, Infrequent, Frequent, Constant), Chest-back pain (None, Relieved by nonnarcotics, Relieved by narcotics, Not relieved by narcotics), JSGS-Q, Do you suffer from regurgitation (Yes/No), Incidence of chronic postsurgical pain |
| --- | --- |

| 6. Role Functioning & Daily Activities | LIADL, SMES, ESCAS, SUPPH |
| --- | --- |

| 7. Cognitive & Existential Well-being | PSQI, SMHSQ, PDI, FACT-HN, M-FACT-HN, FACT-G, FACT, QLQ-INFO25 |
| --- | --- |

**Examples of how interventions were coded into a specific category**

Each questionnaire or measurement instrument was assigned to a single most representative outcome category based on the primary construct assessed or the dominant domain reported in the included studies. Although several instruments, such as the EORTC QLQ-C30 and FACT-G, evaluate multiple HRQL domains including physical, emotional, and role functioning, they were categorised into only one outcome category to prevent duplication.

| **Category** | **Interventions** |
| --- | --- |
| **Surgical** | Thoracoscopy combined with laparoscopy, Traditional radical surgery, Thoracoscopic esophagectomy, Open esophagectomy, Minimally invasive esophagectomy, Radical surgery + reconstruction with gastric tube and lymph node dissection, Transhiatal esophagectomy (THE), Subtotal esophagectomy via VATS, Total gastrectomy, Transthoracic oesophagectomy, Narrow gastric tube reconstruction, Whole-stomach reconstruction, Esophagectomy and gastric tube reconstruction, Jejunal interposition with stomach-preserving esophagectomy, Elective and emergency esophagectomy, Three-incision open esophagectomy, Fast-track surgery, Hybrid open chest and minimally invasive abdomen, Ivor-Lewis surgery, Anastomotic technique (hand-sewn, mechanical), Open surgery, Minimally invasive surgery, Surgery + chemotherapy + education, Surgery + chemotherapy + pamphlet, mERAS protocol after esophagectomy. |
| **Chemotherapy / Chemoradiotherapy** | Cisplatin + paclitaxel chemotherapy, Camrelizumab + radiotherapy, Neoadjuvant chemoradiotherapy + surgery, Concomitant chemoradiotherapy + intraluminal brachytherapy, Concurrent chemoradiotherapy (CCRT) with thalidomide (THAL), Concurrent chemoradiotherapy with cisplatin and 5-fluorouracil, Definitive chemoradiation alone, Docetaxel/5-FU/CDDP (DFP) therapy with/without Rikkunshito, Combination of HDR brachytherapy + EBRT, Late course accelerated hyperfractionated irradiation (LCAF), Cisplatin-based neoadjuvant chemotherapy + Ghrelin, Chemotherapy + radiotherapy, Induction chemoradiation followed by surgery or continuation of chemoradiation, Chemoradiotherapy + nutrition management, Chemoradiotherapy alone, Chemotherapy alone, Tegafur + lentinan, Chemotherapy regimens with capecitabine, paclitaxel, cisplatin. |
| **Stenting and Palliative Endoscopic Therapies** | Self-expanding metal stent (SEMS), Antireflux stent, Metallic stent, Plastic cendoprosthesis, Metal stent + brachytherapy, Covered SEMS with antireflux valve, Hanarostent, Endoscopic sequential treatment (local chemotherapy, RF therapy, iodine-125 implantation), Thermal ablative therapy, Laser therapy, Argon plasma coagulation, Bipolar probe electrocoagulation, Photodynamic therapy, Esophageal bypass surgery, Esophageal dilatation, Combination of stenting and EBRT. |
| **Nutritional Interventions** | Extended preoperative nutritional support + home enteral nutrition, Standard nutritional support, Whole-course nutrition management from the Nutrition Support Team, Specific medical food pre-anticancer therapy, Ghrelin administration as nutritional support, Rikkunshito post-esophagectomy. |
| **Supportive Care and Lifestyle** | Surgery + nursing postoperative pain management strategy, Surgery + comprehensive nursing, Psychological intervention with conventional nursing, Nurse-led telephone supportive care after esophagectomy, Cluster nursing + blood pressure regulation, Psychological nursing intervention, Direct patient education vs. pamphlet, Conventional vs. comprehensive supportive care, mERAS protocol vs. traditional nursing. |
| **Immunotherapy** | Adoptive CIK cell + DC therapy + IMRT, CIK + DC therapy alone, Combination of dendritic cells and cytokine-induced killer cells (immunotherapy) + conventional treatment. |
| **Traditional and Complementary Medicine** | Traditional Chinese medicine alone, Combination of chemotherapy with Chinese medicinal herbs, Î²-elemene-assisted radiotherapy/chemotherapy, Tegafur + lentinan, Rikkunshito administration. |

**Examples of how interventions were coded into a specific category**

Individual interventions were categorised into broader categories of intervention types. Each intervention was assigned to one category to prevent double counting. In instances where an intervention could fit into multiple categories (e.g. studies comparing surgery alone to surgery combined with chemotherapy), the classification was based on the most significant or distinct aspect of the intervention. Thus, these studies were classified under the chemotherapy/chemoradiotherapy category. Additionally, four studies included multiple interventions involving surgery and were categorised under the surgical section.

**Frequency of Use of Questionnaires and Items Across Included Studies**

This figure illustrates the distribution of all questionnaires and the individual items used in the included studies. Each bar represents a specific questionnaire or item reflecting the number of times it was used across the included studies. Outcomes reported only once or twice were grouped into a collective category labelled “Others.”

Documentation of search strategies

University Library search request group

Date: March 2025

Topic/research question: HRQoL in oesophageal cancer

Name of researcher(s): Kenneth Färnqvist

Librarian(s): Emma-Lotta Säätelä & Ingrid Andersson

Databases:

1. Medline (Ovid)
2. Embase (embase.com)

A literature search was performed using the Medline (Ovid) and Embase (embase.com) databases. The last search was conducted on March 5, 2025.

1. Medline

| Interface: **Ovid MEDLINE(R) ALL** content coverage from 1946  Date of Search: March 5 2025  Number of hits: 3,304  Comment: In Ovid, two or more words are automatically searched as phrases; i.e. no quotation marks are needed | Field labels   - exp/ = exploded MeSH term - / = non exploded MeSH term - .ti,ab,kf. = title, abstract and author keywords - adjx = within x words, regardless of order - * = truncation of word for alternate endings - ? = 0-1 letter/number - # = 1 letter/number |
| --- | --- |
| Database(s): **Ovid MEDLINE(R) ALL**1946 to March 04, 2025 Search Strategy:   \| **#** \| **Searches** \| **Results** \| \| --- \| --- \| --- \| \| 1 \| exp Esophageal Neoplasms/ \| 62897 \| \| 2 \| ((esoph* or oesoph*) adj3 (adenocarcinoma* or carinoma* or cancer* or malignan* or neoplas* or tumor* or tumour*)).ti,ab,kf. \| 55739 \| \| 3 \| or/1-2 \| 80557 \| \| 4 \| exp "Quality of life"/ \| 302029 \| \| 5 \| Health status/ \| 93320 \| \| 6 \| Health Status Indicators/ \| 24172 \| \| 7 \| Psychosocial Functioning/ \| 526 \| \| 8 \| exp "Activities of Daily Living"/ \| 127817 \| \| 9 \| Patient Reported Outcome Measures/ \| 17720 \| \| 10 \| exp Adaptation, psychological/ \| 145229 \| \| 11 \| exp Psychological Tests/ \| 359211 \| \| 12 \| "Surveys and Questionnaires"/ \| 614293 \| \| 13 \| Health surveys/ \| 68701 \| \| 14 \| exp Health care surveys/ \| 53486 \| \| 15 \| (QLQ or FACT E or MDASI Eso or SF 36 or EQ 5D or WHOQOL or FACT G or Brief Fatigue Inventory or "Hospital Anxiety and Depression" or Life Orientation Test or IPQ R or IES R or "Assessment of Survivor Concerns").ti,ab,kf. \| 67533 \| \| 16 \| (quality adj2 life).ti,ab,kf. \| 450814 \| \| 17 \| ("Activities of Daily Living" or ADL or functioning or functional status* or health status* or HRQOL or patient report* or PRO? or QOL or self care* or self report* or "sense of coherence" or psychosocial* or psycho social* or well being or wellbeing).ti,ab,kf. \| 1306670 \| \| 18 \| or/4-17 \| 2559421 \| \| 19 \| 3 and 18 \| 4023 \| \| 20 \| limit 19 to (english language and yr="2000 -Current") \| 3304 \|  \|  \| \| --- \| | |

2. Embase

| Interface: **embase.com** content coverage from 1947  Date of Search: March 5 2025  Number of hits: 4,116  Comment: Emtree is the controlled vocabulary in Embase | Field labels   - /exp = exploded Emtree term - /de = non exploded Emtree term - ti,ab,kw = title, abstract and author keywords - NEAR/x = within x words, regardless of order - * = truncation of word for alternate endings - $ = 0-1 letter/number - ? = 1 letter/number |
| --- | --- |
| \| No. \| Query \| Results \| \| --- \| --- \| --- \| \| #19 \| #17 NOT #18 \| 4116 \| \| #18 \| #3 AND #15 AND [english]/lim AND [2000-2025]/py AND ([conference abstract]/lim OR [conference review]/lim) \| 1855 \| \| #17 \| #3 AND #15 AND [english]/lim AND [2000-2025]/py \| 5970 \| \| #16 \| #3 AND #15 \| 6753 \| \| #15 \| #4 OR #5 OR #6 OR #7 OR #8 OR #9 OR #10 OR #11 OR #12 OR #13 OR #14 \| 2391357 \| \| #14 \| 'activities of daily living':ti,ab,kw OR adl:ti,ab,kw OR functioning:ti,ab,kw OR 'functional status*':ti,ab,kw OR 'health status*':ti,ab,kw OR hrqol:ti,ab,kw OR 'patient report*':ti,ab,kw OR pro$:ti,ab,kw OR qol:ti,ab,kw OR 'self care*':ti,ab,kw OR 'self report*':ti,ab,kw OR 'sense of coherence':ti,ab,kw OR psychosocial*:ti,ab,kw OR 'psycho social*':ti,ab,kw OR 'well being':ti,ab,kw OR wellbeing:ti,ab,kw \| 1805504 \| \| #13 \| (quality NEAR/2 life):ti,ab,kw \| 698036 \| \| #12 \| qlq:ti,ab,kw OR 'fact e':ti,ab,kw OR 'mdasi eso':ti,ab,kw OR 'sf 36':ti,ab,kw OR 'eq 5d':ti,ab,kw OR whoqol:ti,ab,kw OR 'fact g':ti,ab,kw OR 'brief fatigue inventory':ti,ab,kw OR 'hospital anxiety and depression':ti,ab,kw OR 'life orientation test':ti,ab,kw OR 'ipq r':ti,ab,kw OR 'ies r':ti,ab,kw OR 'assessment of survivor concerns':ti,ab,kw \| 113728 \| \| #11 \| 'mental disease assessment'/exp/mj OR 'behavior assessment'/exp/mj OR 'general health status assessment'/exp/mj OR 'psychophysiologic assessment'/exp/mj \| 58047 \| \| #10 \| 'psychological adjustment'/exp/mj \| 5491 \| \| #9 \| 'questionnaire'/exp/mj \| 48267 \| \| #8 \| 'patient-reported outcome'/mj \| 21491 \| \| #7 \| 'daily life activity'/mj \| 17920 \| \| #6 \| 'health status indicator'/mj \| 1657 \| \| #5 \| 'health status'/exp/mj \| 99246 \| \| #4 \| 'quality of life'/mj \| 153806 \| \| #3 \| #1 OR #2 \| 123512 \| \| #2 \| ((esoph* OR oesoph*) NEAR/3 (adenocarcinoma* OR carinoma* OR cancer* OR malignan* OR neoplas* OR tumor* OR tumour*)):ti,ab,kw \| 81976 \| \| #1 \| 'esophagus cancer'/exp \| 99357 \| | |

**References**

1. White H, Albers B, Gaarder M, Kornør H, Littell J, Marshall Z, et al. Guidance for producing a Campbell evidence and gap map. Campbell Systematic Reviews. 2020;16(4).

2. Garritty C, Hamel C, Trivella M, Gartlehner G, Nussbaumer-Streit B, Devane D, et al. Updated recommendations for the Cochrane rapid review methods guidance for rapid reviews of effectiveness. BMJ. 2024:e076335.

3. Clark JM, Sanders S, Carter M, Honeyman D, Cleo G, Auld Y, et al. Improving the translation of search strategies using the Polyglot Search Translator: a randomised controlled trial. Journal of the Medical Library Association. 2020;108(2).

4. Dobrescu AI, Nussbaumer-Streit B, Klerings I, Wagner G, Persad E, Sommer I, et al. Restricting evidence syntheses of interventions to English-language publications is a viable methodological shortcut for most medical topics: a systematic review. J Clin Epidemiol. 2021;137:209-17.

5. Bramer WM, Giustini D, De Jonge GB, Holland L, Bekhuis T. De-duplication of database search results for systematic reviews in EndNote. Journal of the Medical Library Association : JMLA. 2016;104(3):240-3.

6. Jansen JP, Fleurence R, Devine B, Itzler R, Barrett A, Hawkins N, et al. Interpreting Indirect Treatment Comparisons and Network Meta-Analysis for Health-Care Decision Making: Report of the ISPOR Task Force on Indirect Treatment Comparisons Good Research Practices: Part 1. Value in Health. 2011;14(4):417-28.

7. Shea BJ, Reeves BC, Wells G, Thuku M, Hamel C, Moran J, et al. AMSTAR 2: a critical appraisal tool for systematic reviews that include randomised or non-randomised studies of healthcare interventions, or both. BMJ. 2017:j4008.

8. Ringsten M, Färnqvist K, Bruschettini M, Johansson M. Inclusion, characteristics and methodological limitations of systematic reviews in doctoral theses: A cross‐sectional study of all universities in Sweden. Cochrane Evidence Synthesis and Methods. 2025;3(1).

9. Sterne JAC, Savović J, Page MJ, Elbers RG, Blencowe NS, Boutron I, et al. RoB 2: a revised tool for assessing risk of bias in randomised trials. BMJ. 2019:l4898.

10. Sterne JA, Hernán MA, Reeves BC, Savović J, Berkman ND, Viswanathan M, et al. ROBINS-I: a tool for assessing risk of bias in non-randomised studies of interventions. BMJ. 2016:i4919.
